# Auditory-Motor Control of Vocal Production during Divided Attention: Behavioral and ERP Correlates

**DOI:** 10.3389/fnins.2018.00113

**Published:** 2018-02-27

**Authors:** Ying Liu, Hao Fan, Jingting Li, Jeffery A. Jones, Peng Liu, Baofeng Zhang, Hanjun Liu

**Affiliations:** ^1^Department of Rehabilitation Medicine, The First Affiliated Hospital, Sun Yat-sen University, Guangzhou, China; ^2^Psychology Department and Laurier Centre for Cognitive Neuroscience, Wilfrid Laurier University, Waterloo, ON, Canada; ^3^Guangdong Provincial Key Laboratory of Brain Function and Disease, Zhongshan School of Medicine, Sun Yat-sen University, Guangzhou, China

**Keywords:** auditory feedback, speech motor control, divided attention, attentional load, working memory

## Abstract

When people hear unexpected perturbations in auditory feedback, they produce rapid compensatory adjustments of their vocal behavior. Recent evidence has shown enhanced vocal compensations and cortical event-related potentials (ERPs) in response to attended pitch feedback perturbations, suggesting that this reflex-like behavior is influenced by selective attention. Less is known, however, about auditory-motor integration for voice control during divided attention. The present cross-modal study investigated the behavioral and ERP correlates of auditory feedback control of vocal pitch production during divided attention. During the production of sustained vowels, 32 young adults were instructed to simultaneously attend to both pitch feedback perturbations they heard and flashing red lights they saw. The presentation rate of the visual stimuli was varied to produce a low, intermediate, and high attentional load. The behavioral results showed that the low-load condition elicited significantly smaller vocal compensations for pitch perturbations than the intermediate-load and high-load conditions. As well, the cortical processing of vocal pitch feedback was also modulated as a function of divided attention. When compared to the low-load and intermediate-load conditions, the high-load condition elicited significantly larger N1 responses and smaller P2 responses to pitch perturbations. These findings provide the first neurobehavioral evidence that divided attention can modulate auditory feedback control of vocal pitch production.

## Introduction

Auditory feedback is critical for the production of proper speech sounds (Hickok et al., 2011). Numerous behavioral studies have demonstrated that speakers compensate for alterations in voice pitch, loudness, and formant frequencies by producing vocal adjustments against the direction of the alterations (Burnett et al., 1998; Jones and Munhall, 2002; Bauer et al., 2006; Purcell and Munhall, 2006; Liu and Larson, 2007; Liu et al., 2007; Macdonald et al., 2010; Mitsuya et al., 2015). This compensatory control process can be modulated by task demands (Natke et al., 2003; Chen et al., 2007) and shaped by language and music experience (Zarate and Zatorre, 2008; Liu et al., 2010; Mitsuya et al., 2013; Behroozmand et al., 2014). Furthermore, patients with Parkinson's disease (PD), Alzheimer's disease (AD), and temporal lobe epilepsy (TLE) produce abnormally enhanced vocal compensations for pitch perturbations (Liu et al., 2012; Chen et al., 2013; Mollaei et al., 2016; Ranasinghe et al., 2017). Thus, understanding the mechanisms that underlie auditory feedback control of speech production is important for the treatment of voice/speech disorders caused by neurological diseases.

Brief auditory feedback perturbations typically evoke rapid compensatory vocal responses with short latencies of ~80–150 ms (Larson, 1998; Burnett and Larson, 2002; Chen et al., 2007; Liu and Larson, 2007). Moreover, speakers are unable to consciously modify their vocal compensations even when told to, suggesting that the feedback-based control of speech production is a reflex-like process (Munhall et al., 2009; Keough et al., 2013). There is evidence, however, suggesting that auditory feedback control of speech production may be subject to attentional control. Previous research has repeatedly shown that attended auditory stimuli elicit larger event-related potentials (ERPs) (Hink and Hillyard, 1976; Stevens et al., 2006) and enhanced brain activity in the auditory cortex (Ahveninen et al., 2006; Johnson and Zatorre, 2006; Sabri et al., 2008) relative to unattended auditory stimuli. These findings suggest that the perception of speech sounds is highly dependent on attention. Similarly, auditory-motor interactions during speech processing can also be facilitated by attention, as reflected by increased left-hemisphere P50 m responses when participants attended to lip-articulated “ba” sounds while their cortical motor lip area was disrupted by transcranial magnetic stimulation (TMS) (Möttönen et al., 2014). In the context of speech motor control, Tumber et al. (2014) reported that participants who were exposed to pitch perturbations during vocalization produced smaller vocal compensations when they actively attended to a rapid serial visual presentation (RSVP) of letters relative to when they passively viewed the RSVP, suggesting that the attentional load of the RSVP task reduced the available attentional resources for the detection and/or correction for production errors. In two other studies conducted in our laboratory (Hu et al., 2015; Liu et al., 2015), participants were asked to attend to pitch perturbations they heard in voice auditory feedback, or attend to flashing lights they viewed during the production of sustained vowels. The results showed that attending to pitch perturbations elicited significantly larger vocal compensations and P2 responses relative to ignoring pitch perturbations (i.e., attending to flashing lights) and passively observing the bimodal stimuli. These neurobehavioral findings can be accounted for by the gain-based theory of selective attention (Hillyard et al., 1998), according to which selective attention increases the gain for neurons involved in auditory-vocal integration which in turn facilitates the detection/correction of voice feedback errors.

It is noteworthy that during daily communication attention is often divided such that auditory feedback can be processed in conjunction with other sensory information simultaneously. The effect of divided attention on auditory-motor integration for voice control, however, is far from clear. Previous studies have shown decreased brain activity in both the auditory and visual cortices but increased brain activity in the lateral frontal regions when attention is divided between auditory and visual stimuli compared to when attention is focused on either auditory or visual stimuli alone (Klingberg, 1998; Loose et al., 2003; Johnson and Zatorre, 2006; Moisala et al., 2015). In one recent ERP study by Getzmann et al. (2016), dividing attention to speech from two speakers led to smaller N1 and P2 responses than focusing attention on speech from one speaker. Likewise, this dual-task interference during divided attention influences auditory-motor control of vocal production. Liu et al. (2015) reported significantly smaller P2 responses when attention was divided to both pitch perturbation and flashing lights as compared to when pitch perturbations were selectively attended and ignored. In addition, dividing attention to the bimodal stimuli elicited significantly larger N1 responses and smaller P2 responses relative to passively observing the bimodal stimuli. These findings suggest that divided attention can modulate the cortical processing of mismatches between intended and actual vocal output.

Certain shortcomings in the study by Liu et al. (2015), however, limit our understanding of how divided attention influences auditory-motor control of vocal production. First of all, although the N1 and P2 responses have been hypothesized to, respectively, reflect the early detection of mismatches between predicted and actual voice auditory feedback and the later cortical activity involved in auditory-motor interaction (Behroozmand et al., 2011; Guo et al., 2016), the observed modulation of N1 and P2 responses in Liu et al. (2015) may instead be the result of attention-driven central auditory processing of pitch feedback errors because vocal compensations did not vary as a function of divided attention. Next, as compared to when participants divided attention to pitch perturbations and flashing lights, N1 responses were significantly larger when participants passively observed the bimodal stimuli whereas remained intact when participants attended to flashing lights while ignoring pitch perturbations. Whether this N1 enhancement was a result of divided attention remains unclear. Finally, given that the lateral frontal regions subserving working memory were recruited during divided attention but not selective attention (Johnson and Zatorre, 2006), Liu et al. (2015) attributed the modulation of cortical N1 and P2 responses to pitch perturbations during divided attention to the interaction between working memory and divided attention. There is at present insufficient evidence, however, to support this hypothesis. Thus, the present study aims to extend results from our previous investigation (Liu et al., 2015) and thereby expand current knowledge about the interaction between divided attention and auditory-vocal integration.

In summary, little is currently known about the effect of divided attention on the auditory-motor control of speech production. In order to address this important question, the present study examined the behavioral and ERP correlates of auditory feedback-based vocal pitch regulation during divided attention. We adapted the previously used paradigm (Liu et al., 2015), during which participants were instructed to attend to pitch perturbations in auditory feedback and red indicator lights on the screen simultaneously while producing sustained vowels. These two sensory stimuli were behaviorally irrelevant and their presentation did not overlap to avoid any interaction between multisensory integration and attention control. In order to vary the attentional resources available for auditory feedback processing during divided attention, we varied the inter-stimulus intervals (ISIs) in the presentation rate of the red indicator lights to impose a low, intermediate, and high attentional load. This paradigm has been successfully used in previous studies of divided attention (Craik et al., 1996; Naveh-Benjamin et al., 2000; Uncapher and Rugg, 2005) and allowed us to compare the neurobehavioral responses to pitch perturbations across the three attentional load levels. In light of our previous findings (Hu et al., 2015; Liu et al., 2015), we hypothesized that divided attention would exert modulatory effects on the neurobehavioral responses to pitch feedback errors during vocal production and that such effects would change as a function of attentional load.

## Materials and methods

### Subjects

Forty native Mandarin-speaking young adults participated in the present study. Eight participants were excluded from the final data pool because of poor data quality. Thus, data from thirty-two participants [21 female and 11 male; mean age and standard deviation (SD): 21.53 ± 2.41 years] entered the final statistical analyses. They were all right-handed, had normal or corrected-normal vision, and reported no history of hearing, speech, language, or neurological disorders. Hearing thresholds were screened at 25 dB HL for octave intervals of 500–4000 Hz. Written informed consent was obtained from all participants. The research protocol that was in accordance with the Code of Ethics of the World Medical Association (Declaration of Helsinki) was approved by the Institutional Review Board of The First Affiliated Hospital at Sun Yat-sen University of China.

### Apparatus

The experiment was conducted in a sound-attenuated booth, where participants' voice and electroencephalographic (EEG) signals were recorded. In order to partially mask the air-born and bone-conducted feedback, we calibrated the acoustic recording system so that the intensity of voice feedback the participant heard was 10 dB SPL higher than that of his/her voice output. During the experiment, participants' voice signals were transduced by a dynamic microphone (DM2200, Takstar Inc.) and sent to an Eventide Eclipse Harmonizer via a MOTU Ultralite Mk3 Firewire audio interface. A custom-developed Max/MSP software program (v.5.0 by Cycling 74) controlled the Harmonizer to pitch-shift the voice signals and sent them to an ICON NeoAmp headphone amplifier. The amplified pitch-shifted voices were presented to participants as attended auditory stimuli through insert earphones (ER1-14A, Etymotic Research Inc.). Two circles representing the blue and red indicator lights were generated by the Max/MSP software program and displayed on the computer screen. The blue indicator light was used to cue the start and end of vocalization, while the flashing red indicator lights were used as attended visual stimuli. Transistor-transistor logic (TTL) control pulses were also generated by this program to mark the onset of the pitch perturbations. The original and pitch-shifted voice signals as well as the TTL control pulses were sampled at 10 kHz by a PowerLab A/D converter (ML880, AD Instruments) and recorded using LabChart software (v.7.0 by AD Instruments).

While recording the voice signals, we collected the EEG signals from 64 sites on the participant's scalp using a Geodesic Sensor Net (Electrical Geodesics Inc.). Scalp-recorded brain potentials were amplified by a Net Amps 300 amplifier that accepts scalp-electrode impedances up to 40–60 kΩ (Z_in_≈200 MΩ; Electrical Geodesics Inc.), digitized at 1 kHz, and recorded using NetStation software (v. 4.5, Electrical Geodesics Inc.). The TTL control pulses that signaled the onset of the pitch perturbations were sent to the EEG recording system via a DIN synch cable. The EEG signals across all channels were referenced to the vertex (Cz) during the online recording. Electrode impedances were maintained at ≤50 kΩ for individual sensors (Ferree et al., 2001).

### Procedure

In the present study, participants were instructed to produce and maintain a steady vocalization of the vowel /u/ at their comfortable pitch and loudness level when the blue indicator light was turned on and terminate their vocalizations when the blue indicator was turned off. During each vocalization, participants heard their voice pitch randomly shifted +200 cents (100 cents = one semitone) while seeing a number of red indicator light flashes on the computer screen. The number of the pitch perturbations ranged from one to five per vocalization. The first pitch perturbation occurred 500–1000 ms after the onset of vocalization, and the succeeding stimuli were presented with an inter-stimulus ISI of 700–900 ms. The red indicator light flashed 1–13 times per vocalization. The first red indicator light began to flash 500 ms after the blue indicator light prompted participants to vocalize, and the succeeding stimuli were presented with three different ISIs: 1400–2000 ms (1400, 1600, 1800, and 2000 ms; low load), 900–1500 ms (900, 1100, 1300, and 1500 ms; intermediate load), and 400–1000 ms (400, 600, 800, and 1000 ms; high load). The onsets of auditory and visual stimuli were asynchronous. The durations of both the red indicator light and pitch perturbation were fixed at 200 ms. Production of ~40 consecutive vocalizations constituted one block, which led to ~100 trials (i.e., pitch perturbation) per condition. The order of the three attentional load conditions was counterbalanced across all subjects.

While producing sustained vocalizations, participants were required to divide their attention to auditory (pitch feedback perturbations) and visual stimuli (flashing red lights) across the three load conditions. An immediate recall test was performed after each vocalization, during which they reported the number of the pitch perturbations that they heard and the number of the red indicator light flashes that they saw. This test ensured that participants attended to the bimodal stimuli as required. Their behavioral performance, as indexed by the percentage of correctly remembered auditory and visual stimuli across the three load conditions, was evaluated and submitted to statistical analyses.

### Data analysis

The event-related averaging technique (Li et al., 2013) was applied to the measurements of the magnitudes and latencies of vocal responses to pitch perturbations using a custom-developed IGOR PRO software program (v.6.0 by Wavemetrics Inc.). First, the voice F_0_ contours in Hertz were extracted from the voice signals using Praat software (Boersma, 2001) and converted to the cents scale using the following formula: cents = 100 × (12 × log_2_(F_0_/reference)) [reference = 195.997 Hz (G3)]. The voice contours in cents were then segmented into epochs of 200 ms before to 700 ms after the onset of the pitch perturbation. All individual trials were carefully inspected using a waterfall procedure and trials with signal processing errors or unexpected vocal stops were rejected from further analyses. Finally, the artifact-free trials were normalized by subtracting the mean F_0_ values in the baseline period (−200 to 0 ms) from the F_0_ values after the perturbation onset and then averaged to generate an overall response. The magnitude of a vocal response in cents was measured as the greatest F_0_ value following the response onset. The latency was defined as the time when the voice F_0_ contours exceeded 2 SDs above or below the pre-stimulus mean following the perturbation onset.

Cortical ERPs to pitch-shifted voice auditory feedback were measured using NetStation software. The EEG data were first band-pass filtered at 1–20 Hz and then segmented into epochs ranging from 200 ms before to 500 ms after the onset of the pitch perturbation. Following an artifact detection procedure, segmented trials with voltage values that exceeded ±55 μv of the moving average over an 80-ms window were rejected from further analysis. Additional visual inspection of all individual trials was performed to ensure that all trials with artifacts were removed. Individual electrodes were determined as bad electrodes if they contained artifacts in more than 20% of the segments, and any file that contained more than 10 bad electrodes was excluded. As a result, 88% of the trials were retained and re-referenced to the average of electrodes on each mastoid. Trials were then averaged and baseline-corrected to generate an overall ERP response for each condition. The amplitudes and latencies of N1 and P2 components (Hawco et al., 2009; Chen et al., 2012) were extracted as the negative and positive peaks in the time windows of 80–180 ms and 160–280 ms.

The magnitudes and latencies of vocal and cortical responses (N1 and P2) to pitch feedback perturbations were analyzed using repeated-measures analysis of variance (RM-ANOVAs) in SPSS (v. 16.0). The magnitudes and latencies of vocal responses were subjected to one-way RM-ANOVAs, in which attentional load (low, intermediate, and high load) was chosen as a within-subject factor. The amplitudes and latencies of N1 and P2 responses from 10 fronto-central electrodes (FC1, FC2, FCz, FC3, FC4, C1, C2, Cz, C3, and C4) were subjected to three-way RM-ANOVAs, including three within-subject factors of attentional load, anteriority, and laterality. Frontal (FC1, FC2, FCz, FC3, FC4) and central (C1, C2, Cz, C3, and C4) electrodes were chosen as an anteriority factor, while lateral left (FC3, C3), medial left (FC1, C1), midline (FCz, Cz), medial right (FC2, C2), and lateral right (FC4, C4) were used as a laterality factor. The Greenhouse-Geisser was used to correct probability values for multiple degrees of freedom when the assumption of sphericity was violated. Effect size was calculated using partial η^2^ to describe the size of differences between the conditions. *P*-values < 0.05 and partial η^2^ > 0.14 (Richardson, 2011) were required to be considered significant.

## Results

### Behavioral performance

**Figure 1:**
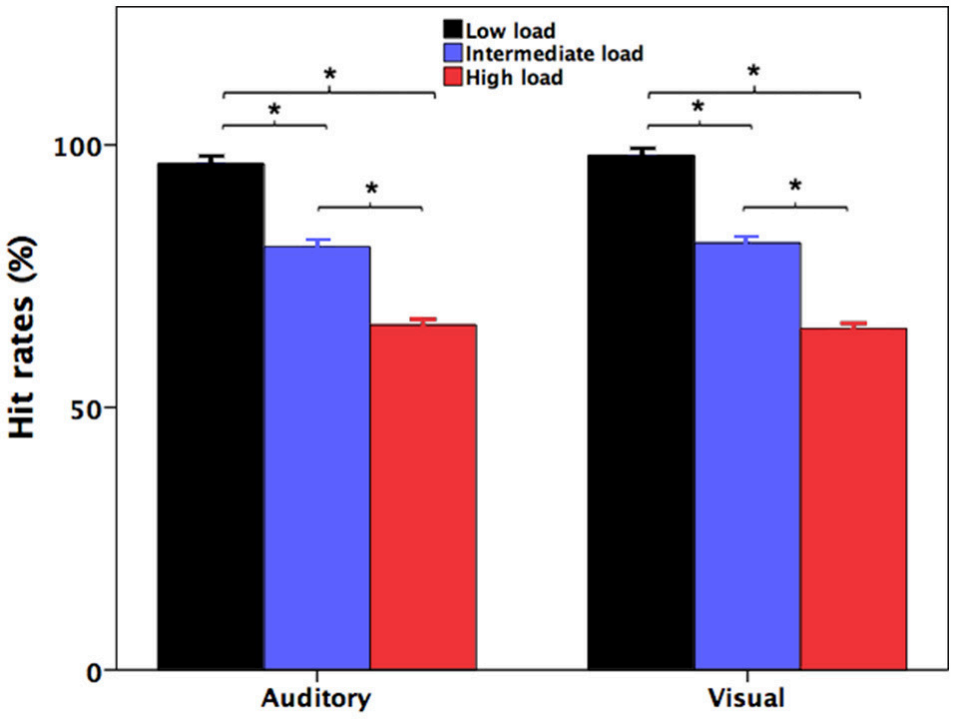
Participants' accuracy at recalling the number of the pitch perturbations (auditory) and the number of the red indicator light flashes (visual) during the low-load (black), intermediate-load (blue), and high-load (red) conditions of divided attention. The asterisks represent significant differences between the load conditions.

Figure 1 shows participants' accuracy at identifying the number of the pitch perturbations (auditory) and the number of the red indicator light flashes (visual) during divided attention as a function of attentional load. Participants' response accuracy for identifying the number of the red indicator light flashes in the high-load condition (65.0 ± 1.0%; mean ± standard errors of the mean throughout unless otherwise indicated) was significantly lower relative to both the intermediate-load (81 ± 0.8%) [*t*_(31)_ = 12.852, *p* < 0.001] and low-load conditions (98.0 ± 0.4%) [*t*_(31)_ = 31.570, *p* < 0.001]. Also, their response accuracy in the intermediate-load condition was also significantly lower than accuracy in the low-load condition [*t*_(31)_ = 19.909, *p* < 0.001]. These results indicate that poorer behavioral performance was associated with faster presentation rate of the red indicator light.

Likewise, participants' accuracy for identifying the number of the pitch perturbations in the high-load condition (65.7 ± 1.1%) was significantly lower than both the intermediate-load (80.6 ± 0.9%) [*t*_(31)_ = 16.880, *p* < 0.001] and low-load conditions (96.4 ± 0.5%) [*t*_(31)_ = 25.790, *p* < 0.001]. Their response accuracy in the intermediate-load condition was also significantly lower than that in the low-load condition [*t*_(31)_ = 10.725, *p* < 0.001]. Therefore, response accuracy for identification of the number of the pitch perturbations was modulated by attentional load created by the different presentation rates of the red indicator lights that participants had to simultaneously count.

### Vocal responses

**Figure 2:**
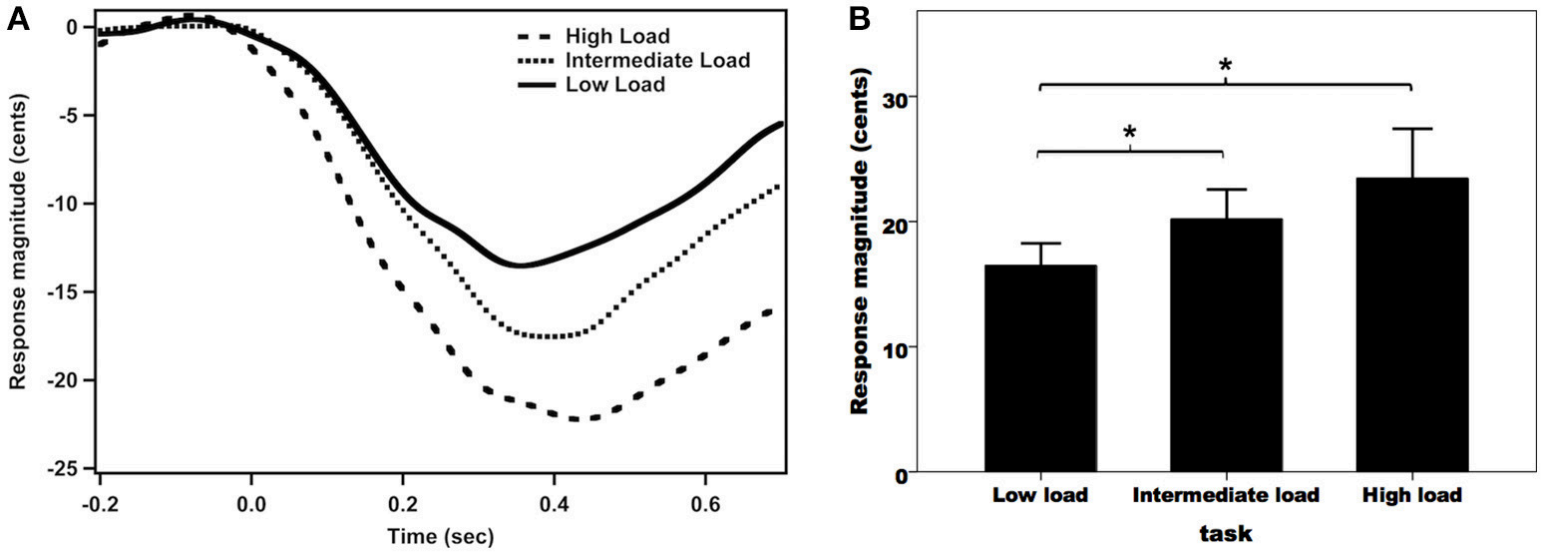
Grand-averaged voice F_0_ contours **(A)** and T-bar graphs of the absolute values of compensatory vocal responses **(B)** to pitch perturbations across the three attentional loads. The thick solid line, the dense dashed line, and the sparse dashed line represent the vocal responses during the low-load, intermediate-load, and high-load conditions of divided attention, respectively. The asterisks represent significant differences between the load conditions.

Figure 2A shows the grand-averaged compensatory voice F_0_ contours in response to pitch perturbations across the three attentional loads. As can be seen, the high-load condition was associated with the largest vocal compensation, followed by the intermediate- and low-load conditions. A one-way RM-ANOVA conducted on the magnitudes of vocal responses revealed a significant main effect of attentional load [*F*_(2, 62)_ = 7.455, *p* = 0.004, partial η^2^ = 0.194]. *Post-hoc* Bonferroni comparison tests showed that the low-load condition (16.5 ± 1.8 cents) elicited significantly smaller response magnitudes than the intermediate-load (20.2 ± 2.4 cents) (*p* = 0.029) and high-load conditions (23.5 ± 4.0 cents) (*p* = 0.015) (see Figure 2B), while the difference between the intermediate-load and high-load conditions did not reach significance (*p* = 0.400). In contrast, the latencies of vocal responses did not vary as a function of attentional load (low-load: 134 ± 13 ms; intermediate-load: 130 ± 13 ms; high-load: 121 ± 11 ms) [*F*_(2, 62)_ = 0.371, *p* = 0.692, partial η^2^ = 0.012].

### ERP responses

**Figure 3:**
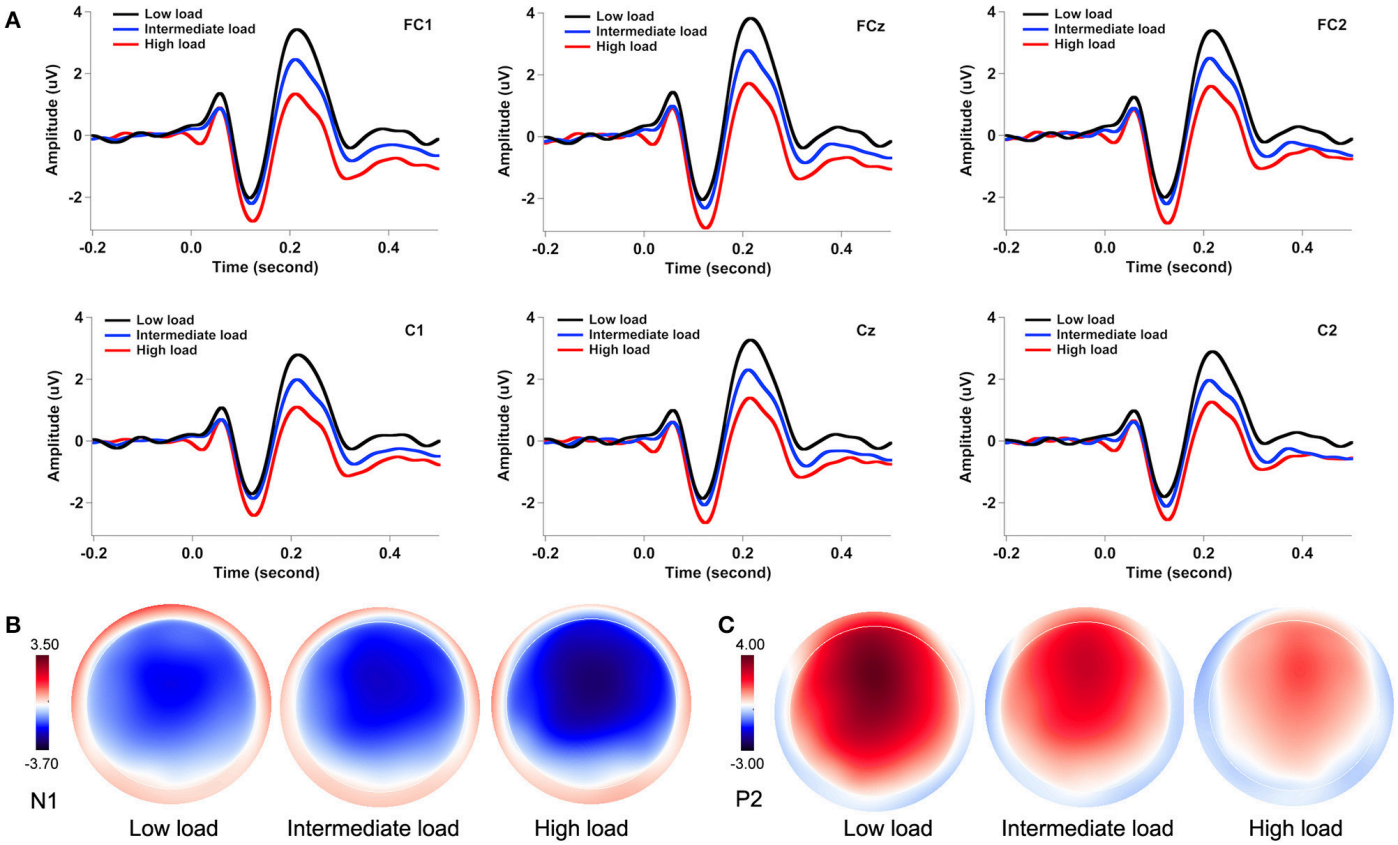
Grand-averaged ERP waveforms **(A)** and topographical distributions of the N1 **(B)** and P2 amplitudes **(C)** in response to pitch perturbations across the three attentional loads. The black, blue, and red solid lines denote the cortical responses during the low-load, intermediate-load, and high-load conditions of divided attention, respectively.

**Figure 4:**
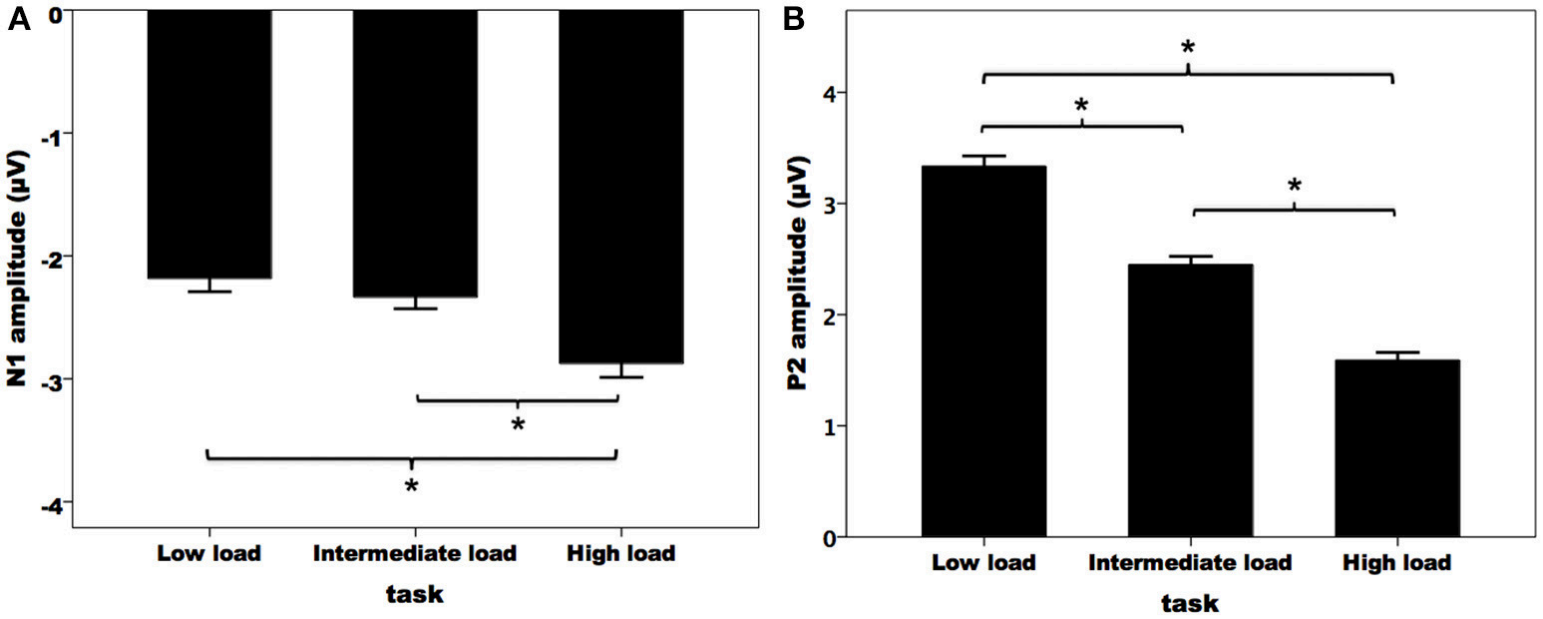
T-bar plots of the N1 **(A)** and P2 **(B)** amplitudes (mean and standard errors) in response to pitch perturbations across the three attentional loads. The asterisks represent significant differences between the load conditions.

Figure 3A illustrates the grand-averaged ERP waveforms in response to pitch perturbations across the three attentional loads. Both the N1 and P2 response appeared to be affected by divided attention, as reflected by increased N1 responses and decreased P2 response with the increasing of attentional load. These effects of divided attention can also be seen in the topographical distributions of the N1 (Figure 3B) and P2 amplitudes (Figure 3C). A three-way RM-ANOVA conducted on the N1 amplitudes revealed a significant main effect of attentional load [*F*_(2, 62)_ = 8.744, *p* = 0.001, partial η^2^ = 0.215]. *Post-hoc* Bonferroni comparison tests showed significantly larger N1 amplitudes (more negative) in the high-load condition relative to the intermediate-load (*p* = 0.009) and low-load conditions (*p* = 0.002) (see Figure 4A), while N1 amplitudes in the low-load and intermediate-load conditions did not differ significantly (*p* = 1.000). Larger N1 amplitudes for the frontal electrodes relative to the central electrodes led to a significant main effect of anteriority [*F*_(1, 31)_ = 16.550, *p* < 0.001, partial η^2^ = 0.348]. There was a significant main effect of laterality [*F*_(4, 124)_ = 8.527, *p* < 0.001, partial η^2^ = 0.216], which was caused by smaller N1 amplitudes for the left medial electrodes relative to the left lateral (*p* < 0.001) and central electrodes (*p* < 0.001).

For the N1 latencies, the main effects of attentional load [*F*_(2, 62)_ = 1.426, *p* = 0.248, partial η^2^ = 0.044] and anteriority [*F*_(1, 31)_ = 2.738, *p* = 0.108, partial η^2^ = 0.081] did not reach significance, whereas a significant main effect of laterality was observed [*F*_(4, 124)_ = 5.756, *p* = 0.008, partial η^2^ = 0.157]. *Post-hoc* Bonferroni comparison tests showed significantly longer N1 latencies for the right lateral electrodes relative to the medial left (*p* = 0.013), medial right (*p* = 0.009), and middle electrodes (*p* = 0.001).

A three-way RM-ANOVA conducted on the P2 amplitudes revealed a significant main effect of attentional load [*F*_(2, 62)_ = 91.495, *p* < 0.001, partial η^2^ = 0.747]. *Post-hoc* Bonferroni comparison tests showed that P2 amplitude was smaller in the high-load condition as compared to the intermediate-load condition (*p* < 0.001) and low-load condition (*p* < 0.001), and P2 amplitude was also smaller in the intermediate-load condition than the low-load condition (*p* < 0.001) (see Figure 4B). The main effect of anteriority [*F*_(1, 31)_ = 46.923, *p* < 0.001, partial η^2^ = 0.602] reached significance, as reflected by significantly smaller P2 responses for the central electrodes relative to the frontal electrodes. There was also a significant main effect of laterality [*F*_(4, 124)_ = 27.066, *p* < 0 .001, partial η^2^ = 0.466] tat was the result of larger P2 amplitudes for the middle electrodes relative to the other electrodes (*p* < 0.02) and larger P2 amplitudes for the medial electrodes relative to the lateral electrodes (*p* < 0.03).

For the P2 latencies, there were no significant main effects of attentional load [*F*_(2, 62)_ = 1.805, *p* = 0.173, partial η^2^ = 0.055] and anteriority [*F*_(1, 31)_ = 0.200, *p* = 0.658, partial η^2^ = 0.006]. However, P2 latencies were modulated as a function of laterality [*F*_(4, 124)_ = 10.174, *p* < 0.001, partial η^2^ = 0.247], as reflected by significantly longer P2 latencies at the lateral right electrodes relative to than the medial left (*p* = 0.002), lateral left (*p* = 0.012), medial right (*p* = 0.003), and middle electrodes (*p* = 0.001).

## Discussion

By asking participants to attend to pitch perturbations in their voice auditory feedback while concurrently performing a low-load, intermediate-load, and high-load visual attention task, the present cross-modal study investigated the auditory-motor processing of vocal pitch errors during divided attention. The behavioral results revealed significantly smaller vocal compensations for attended pitch perturbations in the low-load condition relative to the intermediate-load and high-load conditions. Differential effects of divided attention were observed on the cortical N1 and P2 responses to attended pitch perturbations. The high-load condition elicited significantly larger N1 responses and smaller P2 responses than the intermediate-load and low-load conditions. These findings provide behavioral and neural evidence that divided attention can modulate the auditory-motor processing of vocal pitch errors.

In a previous study by Liu et al. (2015), we showed that dividing attention between pitch perturbations and flashing lights elicited significantly larger N1 responses and smaller P2 responses to pitch perturbations relative to passively observing the bimodal stimuli. In the present study, we found that both N1 and P2 responses to pitch perturbations were differentially modulated by divided attention, with larger N1 and smaller P2 responses elicited by higher attentional loads. These findings add further support to the idea that these two ERP components play different roles in the cortical processing of voice pitch regulation (Behroozmand et al., 2011; Hu et al., 2015; Guo et al., 2017). As important, increased load of divided attention elicited significantly enhanced vocal compensations for pitch perturbations. These findings provide the first behavioral evidence for the modulatory effects of divided attention on auditory feedback control of vocal production. Note that the vocal compensations between the intermediate-load and high-load conditions were not significantly different, nor were the differences of N1 amplitudes between the intermediate-load and low-load conditions significant. Nevertheless, the high-load condition elicited significantly larger vocal and N1 responses and smaller P2 responses than the low-load condition. Thus, the modulatory effect of divided attention on auditory-vocal integration appears to be subject to the degree of attentional load.

Given that attentional capacity is limited (Cowan et al., 2005), one might predict that increasing the presentation rate of the red indicator light flashes would produce increased demands on attention, which would in turn reduce the attentional resources available for identification of the number of pitch perturbations. The reduced attentional resources allocated to pitch feedback errors during the high-load vs. low-load condition should lead to decreased vocal compensations and cortical P2 responses, since focused attention elicits enhanced vocal and cortical P2 responses to pitch perturbations (Tumber et al., 2014; Hu et al., 2015; Liu et al., 2015). Paradoxically, however, the high-load condition elicited enhanced N1 responses and vocal compensations but suppressed P2 responses relative to the low-load condition. An important question comes from our findings then: what are the possible mechanisms underlying these differential neurobehavioral effects of divided attention on the auditory-motor processing of vocal pitch regulation?

One possible account is that these modulatory effects may reflect the interaction between working memory and divided attention in auditory feedback control of speech production. This interpretation is motivated by the fact that working memory is required to store and process multiple independent sensory stimuli during divided attention (Fagioli and Macaluso, 2009; Santangelo and Macaluso, 2013). The prefrontal cortex, which has been implicated in subserving working memory (Curtis and D'Esposito, 2004), is additionally recruited or more active during divided attention as compared to selective attention (Loose et al., 2003; Johnson and Zatorre, 2006; Moisala et al., 2015). Furthermore, brain regions that are involved in working memory are more active when load is increased during divided attention tasks (Uncapher and Rugg, 2005; Santangelo and Macaluso, 2013; Oren et al., 2016). For example, Oren et al. (2016) asked participants to watch movies while simultaneously detecting whether a string of letters was a word or pseudo-word, during which attentional load was manipulated by making the lexical decision task easy and hard. As compared to the low-load condition, the high-load condition was associated with increased activation of the prefrontal cortex (Oren et al., 2016). In another study, Santangelo and Macaluso (2013) required participants to monitor both the object and location of the items. They found that increasing the load of divided attention led to a linear increase in brain activity in the intraparietal sulcus, a brain region that has been activated consistently in working memory studies (Majerus et al., 2007; Harrison et al., 2010). These findings suggest that divided attention and working memory may share a capacity-limited pool of neural resources (Santangelo and Macaluso, 2013). Returning to the present study, it is reasonable to hypothesize that increasing the presentation rate of the red indicator light flashes led to the allocation of more working memory resources to the online processing of both the red indicator light flashes and pitch feedback perturbations.

Along similar lines, recent evidence has shown the effects of working memory on auditory-motor integration for vocal pitch regulation. For example, Guo et al. (2017) reported that enhanced N1 responses in the left middle and superior temporal gyrus, and suppressed P2 responses in the left middle and superior temporal gyrus, inferior parietal lobule, somatosensory cortex, right inferior frontal gyrus and insula were elicited by a delayed match-to-sample (DMS) task that required participants to indicate whether the pitch perturbations they heard during vocalizations in test and sample sequences matched or not. And a significant positive correlation between improved working memory capacity and enhanced P2 responses was found for participants who underwent a training based on a digit-span backward (DSB) paradigm (Li et al., 2015). Considering that precise representations of auditory working memory information can be stored in the auditory cortex (Scott et al., 2014; Huang et al., 2016), enhanced N1 responses in the auditory regions reflect an allocation of more auditory working memory resources to the detection of mismatches between predicted and actual feedback during vocal production. Significant demands on working memory for the storage of pitch perturbations, however, reduce the availability of working memory resources for the auditory-motor transformations, as reflected by suppressed P2 responses in the fronto-parietal regions. In light of this account and the above overlapping hypothesis of divided attention and working memory, our findings of enhanced N1 responses and suppressed P2 responses with increasing attentional load may reflect the engagement of working memory in divided attention, suggestive of increased working memory resources available for the detection of pitch feedback errors but decreased working memory resources available for the auditory-motor transformations. This speculation is supported by one study by Uncapher and Rugg (2005) that required participants to judge whether the words on the screen represented a living or a nonliving thing while attending to an easy and hard auditory task. They found increased activity in the middle occipital cortex and fusiform gyrus and decreased activity in the fronto-parietal regions during the hard vs. easy auditory task.

In addition to the modulation of cortical N1 and P2 responses, we also found enhanced vocal compensations for pitch perturbations in the intermediate-load and high-load conditions relative to the low-load condition. Interestingly, there was also a significant increase of vocal compensations for pitch perturbations in the DMS task that engaged working memory (Guo et al., 2017). Moreover, participants who received extensive auditory working memory training based on a frequency-pattern recognition (FPR) paradigm produced suppressed vocal compensations that were significantly correlated with improved working memory capacity, and enhanced P2 responses in the left middle frontal gyrus, inferior parietal lobule, right inferior frontal gyrus, and insula (Guo et al., 2017). These regions are not only involved in working memory but also in inhibitory control (Aron et al., 2004; Barber et al., 2013; Chmielewski et al., 2017), an important cognitive function that depends on the amount of working memory resources to inhibit reflex-like behavioral responses (Barber et al., 2013; Chmielewski et al., 2015). It is thus suggested that working memory can inhibit compensatory vocal adjustment to prevent vocal production from being excessively influenced by auditory feedback (Guo et al., 2017). In light of these findings, enhanced vocal compensations with increasing attentional load observed in the present study can be accounted for as a result of impaired inhibitory control processes caused by reduced working memory resources for the auditory-motor transformations as reflected by suppressed P2 responses.

It should be noted, however, that our interpretation of the interaction between divided attention and working memory in auditory-vocal integration is speculative. For example, working memory was not directly measured or specifically manipulated in the present study. In addition, whether the observed changes in the N1 and P2 responses to pitch perturbations across the attentional loads received contributions from the neural substrates involved in auditory working memory is unknown due to lack of knowledge about the neural generators of these two ERP components. Future neuroimaging experiments, where participants divide attention to different sensory stimuli while maintaining their specific features (e.g., category, location, etc.,) in working memory, should be conducted to verify our speculation.

In summary, the present cross-modal study investigated the behavioral and neural correlates of auditory-motor integration for vocal pitch regulation during divided attention. The results revealed enhanced vocal compensations for pitch perturbations, enhanced N1 responses, and suppressed P2 responses with increasing load of divided attention, providing neurobehavioral evidence that divided attention can exert top-down influences on auditory feedback control of speech production. Considering the involvement of working memory in divided attention for the storage and maintenance of multiple sensory information (Johnson and Zatorre, 2006; Johnson et al., 2007; Santangelo and Macaluso, 2013), our findings may reflect the contribution of working memory to auditory-vocal integration during divided attention.

## Author contributions

HL and BZ: Designed the experiment; YL, HF, JL, and PL: Performed the experiment and analyzed the data; YL, JJ, BZ, and HL: Interpreted the results and wrote the manuscript. All authors read and approved the final manuscript.

### Conflict of interest statement

The authors declare that the research was conducted in the absence of any commercial or financial relationships that could be construed as a potential conflict of interest.
